# Calibration Method for Monitoring Hygro-Mechanical Reactions of Pine and Oak Wood by Acoustic Emission Nondestructive Testing

**DOI:** 10.3390/ma13173775

**Published:** 2020-08-26

**Authors:** Chiara Bertolin, Lavinia de Ferri, Filippo Berto

**Affiliations:** Department of Mechanical and Industrial Engineering, Norwegian University of Science and Technology, 7491 Trondheim, Norway; lavinia.de.ferri@ntnu.no (L.d.F.); filippo.berto@ntnu.no (F.B.)

**Keywords:** acoustic emission, calibration method, pine, oak, crack propagation, elastic energy, proven fluctuation

## Abstract

The main issue of wood is its sensitivity to Relative Humidity (RH) variations, affecting its dimensional stability, and thus leading to crack formations and propagations. In situ structural health monitoring campaigns imply the use of portable noninvasive techniques such as acoustic emission, used for real-time detection of energy released when cracks form and grow. This paper proposes a calibration method, i.e., acoustic emission, as an early warning tool for estimating the length of new formed cracks. The predictability of ductile and brittle fracture mechanisms based on acoustic emission features was investigated, as well as climate-induced damage effect, leading to a strain-hardening mechanism. Tensile tests were performed on specimens submitted to a 50% RH variation and coated with chemicals to limit moisture penetration through the radial surfaces. Samples were monitored for acoustic emission using a digital camera to individuate calibration curves that correlated the total emitted energy with the crack propagation, specifically during brittle fracture mechanism, since equations provide the energy to create a new surface as the crack propagates. The dynamic surface energy value was also evaluated and used to define a Locus of Equilibrium of the energy surface rate for crack initiation and arrest, as well as to experimentally demonstrate the proven fluctuation concept.

## 1. Introduction

Mechanical damage in materials is procured under the action of stress and deformation. During laboratory tests, a continuously supplied tensile load to a material sample provides energy to the material, and the specimen continuously stores this energy until the ultimate energy storage capacity is reached and energy release occurs [1]. It is well known that the conditions under which a crack can grow when applied a stress (*σ*^2^ in Equation (1)) can be explained by Equation (1) correlating the elastic energy of the material (*E_El_*) to the square of the crack length (*l*^2^) proportional to the inverse of the elasticity modulus (*Y*) as follows:

(1) $$E_{El}∼\frac{l^{2}\sigma^{2}}{Y}$$

The driving force which tends to enlarge the crack can be also defined as the rate of change of strain energy over the crack length. The measure of this driving force is the stress intensity factor taking into account both the applied stress and the geometry of the sample under consideration. The conditions under which a crack can propagate occur when the elastic energy released by the material is higher than the energy required to form a plastic zone or, similarly, when the energy release rate exceeds the critical strain, a characteristic value for each material. It is at the tip of the crack, in this plastic zone, that the stress exceeds the elastic limit of the material. Therefore, it is this plastic zone that effectively controls crack propagation.

In [2], in additional ductile samples, it was highlighted that the greater part of the crack growth, i.e., the majority of the progression of the plastic zone, goes ahead with low acoustic emission (AE) activities.

An acoustic emission is a transient elastic wave caused by the rapid release of energy from specific sources within a material [3]. These waves propagate when unbalanced forces are present. Such condition is satisfied when sudden plastic strains or rapid formation of a new surface in stressed material occur. Brittle fracture illustrates such a pattern, i.e., a sudden creation of new surfaces with the close material (plastic blunting) accelerating in the direction of the now-unbalanced tractive forces through an elastic wave propagation. The energy conversion during crack growth, however, involves both ductile and brittle phenomena as described next.

Ductile fracture in a material is a complex process, due to several phenomena such as dislocation, nucleation, and motion which take place in the neighborhood of the crack tip. In terms of total energy released by the material during the phenomenon, it constitutes a higher percentage of energy spent for the mechano-chemical process in plastic deformation (ca. 75%), as compared with ca. 10–15%, i.e., the surface energy used in disbonding the single layer of atoms that creates the new cracked surface, as stated in [2]. Excess energy is expected to be released in a form different from the elastic one (detectable as an AE event). Such excess energy is, in fact, emitted mostly as heat/radiation of very low amplitude and wide bandwidth frequency and is completely used for steadily progressing the large area of plastic deformed material, as the crack progresses very slowly with quite a stable fracture mechanism (i.e., “initial” crack mechanism). Within this mechanism of progression, the AE energy can only be released at the edge of the extended plastic zone, which is a very limited region. Overall, the assessment of elastic energy detectable with AE released by a ductile material is low (ca. 10% of the total energy), whereas its resistance to crack propagation is high, meaning that the material is less vulnerable.

In contrast, during the brittle fracture phenomenon, the plastic zone is low and not very extended, and the energy required to drive a crack ahead is not high (ca. 15% of the total energy). In such a case, the total excess energy is emitted rapidly and suddenly, and therefore the crack formation is sudden (and not steadily) progressing. Therefore, most of the released energy coincides with the detected acoustic emissions (ca. 75% of the total energy). This has been confirmed in the literature by the detection of higher acoustic emission counts in brittle versus ductile materials [2]. In the overall assessment, the energy released during crack growth of brittle fracture mechanism is proportional to load decrements.

The main objective of this work is to propose a calibration method that uses the acoustic emission non-destructive technique (AE-NDT) as an early warning tool for detecting crack propagation in wood materials used during in situ structural health monitoring campaigns. AE is, in fact, a well-known non-destructive monitoring technique widely employed for laboratory tests [4,5,6,7,8] but still rarely used for in situ structural health monitoring purposes, particularly of architectural structures [9,10,11,12]. Application for monitoring wood-based structures are completely lacking in the literature and this study intends to fill such a gap since it is part of a wider research project funded by the Norwegian Research Council (i.e., SyMBoL-Sustainable Management of Heritage Building in a Long-term Perspective Project) involving on-site monitoring of wood-based architectural structures by means of AE.

Specifically, this contribution investigates predicting different types of fracture mechanisms, i.e., ductile or brittle, as well as, a type of strain-hardening behavior in wooden samples, by looking at the acquired AE data with respect to the load history during the tensile tests.

The mechanical properties of several wood species have been widely investigated in the literature, sometimes coupling the classical mechanical tests with AE. For example, DeBaise et al. [13], in 1966, proposed one of the earliest works on monitoring of wood fracture tests by means of AE for detecting energy released during fracture. The same kind of approach was also used by Schniewind et al. [3] who investigated the formation of stress-induced defects due to the drying process which had been monitored in different studies by AE [14] in order to develop optimized systems.

Concerning monitoring of stresses induced by external loads, Ansell [15] tested three softwoods and found that the shape of the AE stress curve was influenced by the ratio between early- and latewood. Sato et al. [16] distinguished between formation of macro cracks across annual rings, corresponding to fast AEs, and micro cracks related to slow AEs. Reiterer et al. [17] studied the Mode I fracture behavior of various wood species and highlighted that AE counts recorded before reaching the maximum force value are higher for softwoods than for hardwoods. This was interpreted by the authors as confirmation of the ductile character of softwoods with the formation of a process zone with more micro cracks. It was also indicated that macro crack formation was distinguished by propagation from the shape of the cumulative counts and amplitudes. An interesting work by Ando et al. [18] investigated the strength characteristics of new and old woods and evaluated the possibility of reusing old timbers for construction purposes. The analysis of AE features suggested that old wood underwent the final fracture after a long period of stable propagation of the cracks, on the contrary, the new wood failed after almost no period of stable propagation. This behavior, for which no definitive explanation was proposed by Ando et al., resembles the strain-hardening effect observed in this paper.

However, literature is lacking on the distinction of ductile and brittle states by AE, as well as on the analysis of features from the perspective of the already mentioned strain-hardening effect. The present paper aims to provide a solid basis for further systematic investigations.

The appropriate data analysis approach to obtain calibration curves that correlate the crack growth mechanism with the AE recorded energy has been driven by the following: (1) our understanding of AE behaviors during ductile-like (DL) and brittle-like (BL) mechanisms in tensile tests of wood macro structure constituted by early- and latewood and (2) the separation of load increase and decrease steps in tensile tests conducted with a universal testing machine on treated and untreated oak and pine simil-CT samples.

## 2. Materials and Methods

**Figure 1:**
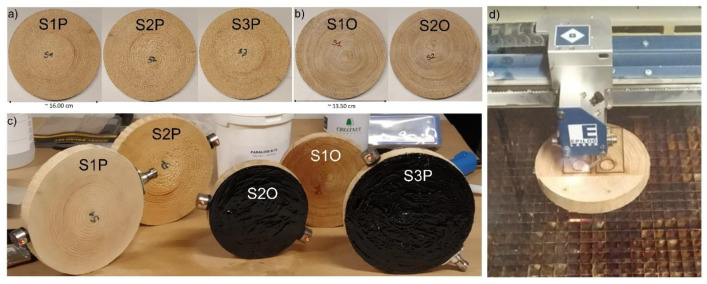
Wood slices acclimatized in climate chamber. (**a**) Pine slices; (**b**) Oak slices; (**c**) Pine and oak slices coated with chemicals; (**d**) Specimen creation method. Names as reported in Table 1.

**Figure 2:**
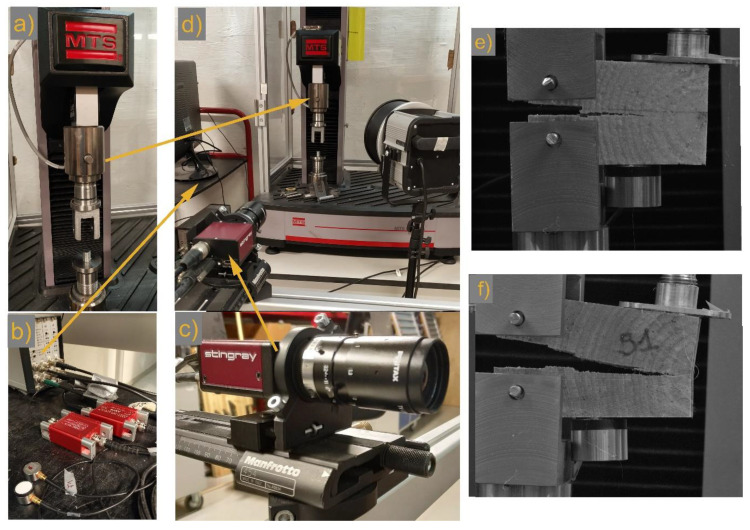
Experimental setup used to conduct the splitting tests on the pine and oak simil-CT samples. (**a**) Universal testing machine (UTM) (MTS producer) with the specially created grippers securing the samples as in (**e**,**f**); (**b**) AE system with the pre-amplifiers and the piezoelectric VS900-M sensors; (**c**) F504B Stingray digital camera synchronized with the UTM; (**d**) Overview of the experimental setup explained in (**a**–**c**). (**e**) Specimen S2.1P; (**f**) Specimen S1.1O, both with AE sensors, i.e., on the top of the specimens the Glazer sensor in its holder, and on the bottom of the specimens, the Vallen VS900-M sensor. Names of the samples as reported in Table 1.

Seventeen fracture tests were conducted and twelve of the tests were considered to obtain the calibration curves for use of AE as an early warning tool for crack propagation estimation. The geometries of the tested simil-CT samples are reported in Table 1 together with indications of the deposited surface treatments. Pine Scots and Oak wood slices were cut in the radial-tangential (RT) direction (Figure 1) and were conditioned in a climate chamber (HPP-IPPPlus, Memmert GmbH + Co. KG, Schwabach, Germany) during an initial acclimatization period at 80% before being treated on two radial surfaces with coating materials consisting of the following: (i) Reinassence microcrystalline wax deposited by means of a spatula (S1.x); (ii) a 40% (*w*/*v*) solution of Paraloid B72 (Phase) in acetone (Sigma-Aldrich, 99.9%) deposited by brush on cellulose sand seal spray (CS) (Verktøy AS-CS), commonly employed in the preparation of wooden materials in order to avoid water capillary rise (S2.x); (iii) tar, deposited by brush (S3.x). Successively, slices were kept at 30% RH in order to create RH-induced stresses in the slices, since treatments are assumed to force moisture movements only through the lateral surface. The surface coatings represented a constraint for the material already prone to shrinkage phenomena and increased the probabilities of failure (formation of macro cracks). Simil-CT samples were cut from such slices for testing (see Figure 1e). Additionally, standard specimens (ST.x), i.e., uncoated, were tested. For more information, please read [19,20]. The effect of RH% variations on the slices was monitored by means of AE and a camera (see Figure 2b–e and explanation below).

During the tensile tests, the samples were hooked by means of specially created grippers to the universal testing machine UTM (MTS company, Eden Prairie, MN 55344 USA, see Figure 2a,d–f) for the load transmission (load cell of 5 kN). During the tensile tests, a very slow crosshead displacement rate of 1.5 mm/min was used. This low value of displacement was selected as it provided the best emission statistic capability limiting the strong oscillations in the force, as well as in the AE signals, during the initial phase of the decay before the mechanical inertia [21].

**Figure 3:**
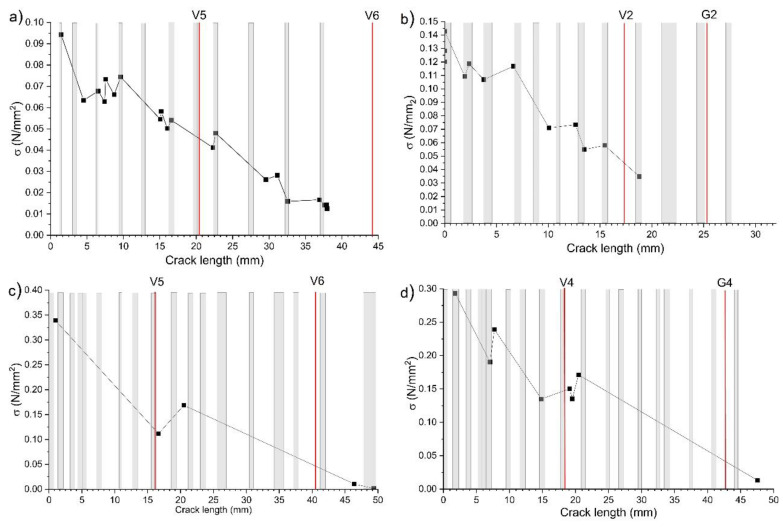
Crack length (mm)/σ (N/mm^2^) curves obtained for 4 of the tested samples. (**a**) STP1; (**b**) SP2.2; (**c**) STO1; (**d**) SO1.1. Red lines designate the position of the AE sensors. V, Vallen; G, Glaser. Grey areas represent the thickness of tree rings (dark areas).

All the UTM stress and strain curves showed a typical pattern with maximum load, and then secondary loads peaks (relative maxima) preceded by load secondary minima. The fracture tests were recorded continuously using the F504B Stingray camera, presented in Figure 2c, acquiring 5 frames/s for the assessment of the crack length propagation at each specific relative maximum and minimum. Figure 3 reports the common sequence of stress versus crack length propagation peaks as obtained by the UTM and the camera. In the plots, the early- and latewood pattern is also highlighted with grey areas representing the thickness of the tree rings and vertical red lines showing the location of the AE sensors, coupled to the samples by means of hot glue. This coupling method has been previously tested on pine and demonstrated to be the an efficient medium to obtain good AE transmission and mounting facilitation. In addition, the quality of the AE sensor coupling was checked before starting the fracture test through the pencil lead break (PLB) method [22].

As previously indicated, in parallel with the UTM and the camera, the AE AMSY-4 (Vallen) system was synchronized during the acquisition for a data comparison during the analysis of the results. The AE AMSY-4 system recorded in real time the acoustic events emitted during the fracture processes using a combination of Vallen (VS900-M) and Glaser (point contact KRNBB-PC) sensors. Concerning the formers, each AE channel was equipped with a VS900-M sensor (frequency operating range = 100–900 kHz) in line with an AEP5 signal preamplifier (2.5 kHz to 2.4 MHz). The rearm time and duration discrimination time were set to 3.2 and 1.6 ms, respectively, while pre- and post-trigger times were maintained equal to 0.2 and 0.4 ms, respectively. The signal sampling rate used to calculate the primary AE parameters was 10 MHz, whereas a value of 1 MHz was used to record the transient signal. Once the first peak was acquired, amplitudes (i.e., maximum value of the first amplitude peak) were integrated to obtain the amplitude values to be verified in the attenuation models presented here. This approach was also used very recently by Zhang et al. [23], and effectively reduces the disturbances caused by the effects of reflection and refraction of AE waves on the results of amplitude attenuation. The Glaser sensors (KRNBB-PC point-contact sensor, frequency operating range = 20 kHz to 1 MHz) were coupled with the same kind of AEP5 signal preamplifier used for the VS900-M sensors. The rearm time and duration discrimination time were both set to 0.4 ms, while the pre-trigger time was kept at 0.2 ms. In addition, in these cases, the signal sampling rate used to calculate the primary AE parameters was 10 MHz, whereas a value of 1 MHz was used to record the transient signal. The selection of the sensors was not by chance, i.e., the same AE sensors ID (cables and amplifier) was utilized during the monitoring of the oak and pine while conditioned in a climate chamber during a previous acclimatization period before the preparation of the simil-CT samples. Sensors VS900-M V5 and V6 were used to test the fracture mechanism of the standard simil-CT reference samples, which had been kept at room condition instead during all the previous acclimatization periods. This work analyzes the AE signal detection of the Vallen sensors only.

### Acoustic Emission and Data Analysis

As anticipated in the Introduction section, acoustic emission (AE) is defined as the energy released during (micro or macro) displacement which occurs in an object (specimens or monitored structure) that is experiencing a deformation [24] induced by a load. For this reason, this technique is particularly useful for tracing physical damages. In particular, brittle cracking events, which are characterized by a rapid rearrangement of internal stresses, lead to the release of energy in the form of transient elastic waves (burst signals with quite short duration and high amplitude) propagating through the material and recordable by means of piezoelectric sensors [25]. Such sensors can detect ultrasonic elastic waves in the frequency range between 1 kHz and 1 MHz [26] and only events (hits) with amplitude values higher than the set threshold (both in dB). In addition to the amplitude of the peak, the following two AE parameters belonging to the time domain can be considered: energy in arbitrary unit (A.U.), i.e., the elastic energy released during single AE events (mathematically defined as the area enclosed by the waveform envelope) and counts (pure number), i.e., the number of times the AE wave crosses the set threshold.

In the frequency domain, two other parameters are defined as follows: center of Gravity (CoG) of the frequency spectrum, reported in Equation (2) as:

(2) $$CoG=\frac{\Sigma \left(magnitude\times frequency\right)}{\Sigma magnitude}$$

and the peak of frequency, i.e., the frequency at which the maximum magnitude in the power spectrum occurs. All these parameters are studied in the present work to characterize the AE signal emitted during DL and BL fracture mechanisms.

The methodological approach used to discriminate between DL or BL crack growth mechanisms is based on the analysis of the AE data acquired during load increase and decrease episodes, respectively. Such intervals were determined looking at the load (N)/time (s) curves obtained by the three coupled instruments, i.e., the UTM, the digital camera, and the AE (see the overview of the used experimental setup in Figure 2).

A load increase episode is defined as the jump in load that occurs between a minimum (i-th peak in the stress and crack propagation curve, as in Figure 3) and its following maximum (i + 1-th peak); while a load decrease episode is defined as the jump in load between a maximum (i − 1-th peak) and its following minimum (i-th peak). Once individuated, all the “sensitive points” on the curves (maxima and minima), the load (N) and time (s) values of all signals recorded by the UTM in each interval (i.e., in each load increase and load decrease interval, respectively) were added up to create a cumulative series for the two modes (DL corresponding to load increase and BL corresponding to load decrease). The same analysis was carried out on AE features (amplitude, energy, and counts), as well as on the load (N) and time (s) values registered by the AE system during each fracture test. This approach distinguished between the “acoustic fingerprint” characteristics of ductile-like (DL) and brittle-like (BL) behavior in the wood. Successively, images, which were collected by the camera at times corresponding to each “sensitive point”, were selected and analyzed using the Surfer 9 software in order to measure the crack length in mm (reported on the *x*-axis of Figure 3). The crack growing in each load increase and decrease step was evaluated and cumulative series were obtained separately for the two modes.

## 3. Results

### 3.1. Acoustic Emission (AE) Data Characteristics of Ductile State

**Figure 4:**
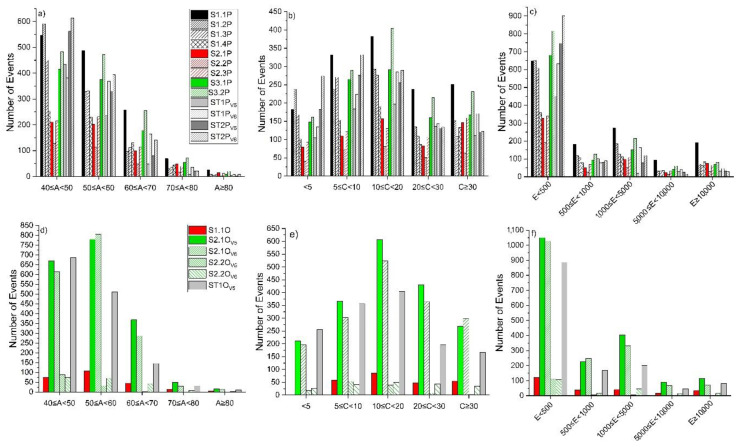
Distribution of AE parameters in ductile-like (DL) state. (**a**) Amplitude, pine; (**b**) Counts, pine; (**c**) Energy, pine; (**d**) Amplitude, oak; (**e**) Counts, oak; (**f**) Energy, oak.

**Figure 5:**
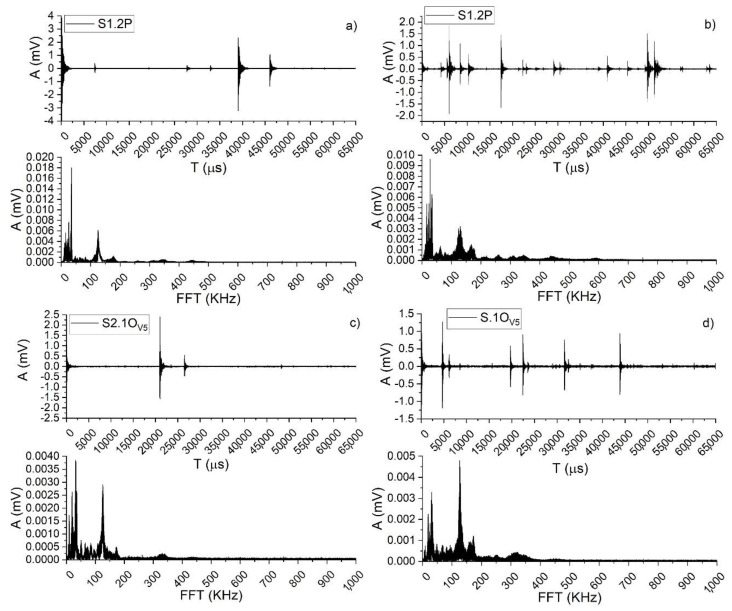
Frequency characteristics of AE features from samples S1.2P (**a**,**b**) and S2.1O_V5_ (**c**,**d**). (**a**) Top, waveform of a signal collected during the brittle state on S1.2P and bottom, FFT of the above AE signal; (**b**) Top, waveform of a signal collected during the ductile state on S1.2P and bottom, FFT of the above AE signal; (**c**) Top, waveform of a signal collected during the brittle state on S2.1O_V5_ and bottom, FFT of the above AE signal; (**d**) Top, waveform of a signal collected during the ductile state on S2.1O_V5_ and bottom, FFT of the above AE signal.

The ductile-like state, in the analyzed samples, was studied after grouping the AE events emitted during the sequence of load increase only. The database representative for these types of events was analyzed looking at the following: (1) the distribution of the characteristic parameters of amplitude, energy, and counts within the five sorter ranges on the x-axes shown in Figure 4 and (2) the frequency spectrum in terms of peak of frequency and center of gravity, shown in Figure 5.

From the analysis of the plots (Figure 4), it emerged that most of the signals recorded for pine samples fall in the classes lower than 50 dB and 500 A.U. for amplitude and energy, respectively. When considering the oak specimens, the majority of the signals fall in the amplitude group including signals between 50 and 60 dB, but again the most populated energy range is the class with events with less than 500 A.U. From the literature [23,27] it is known that real AE signals have low amplitude emission with short duration; in addition and as introduced in Section 1, it is expected that excess energy is emitted during a DL mechanism, mostly as heat/radiation of very low amplitude [1] and wide bandwidth frequency [27].

The frequency domain features (reported in Figure 5) are less dependent on threshold settings than the time domain features. In real AE signal, the detected energy increases with a decrease in the center of gravity (*CoG*) of the frequency, very likely because larger source events take longer to complete, as stated in [27]. As reported in [28], a peak in the distribution of medium frequency (110–170 kHz) accompanies the whole process of damage evolution that is persistent and stable (see FFT in Figure 5). Since the beginning, a large number of middle frequency signals only appear in the early stage of fracture damage. Then, in these early stages of damage evolution (DL fracture, before crack initiation) high frequency (i.e., 290–370 kHz) signals, possibly related to the failure mechanism of cellulose fibers, are more spread than in BL fracture (Figure 5b in respect to Figure 5a). Studies that referred to tensile tests on composites [29] have indicated that peaks in the frequency range of 130–200 kHz were highly influenced by the orientation of the fibers, thus, possibly related to fiber breakage. In addition, Ahn and Nam [30] confirmed that frequencies near 450 kHz were detected from low to high loads on all displacements (i.e., both during DL and BL mechanisms) as this frequency range was representative of two kinds of mechanisms, i.e., the first caused by dislocation and occurrence of slip due to stress concentration applied to the crack tip axis, and the latter caused by the elastic signal.

### 3.2. AE Data Characteristics of the Brittle State

**Figure 6:**
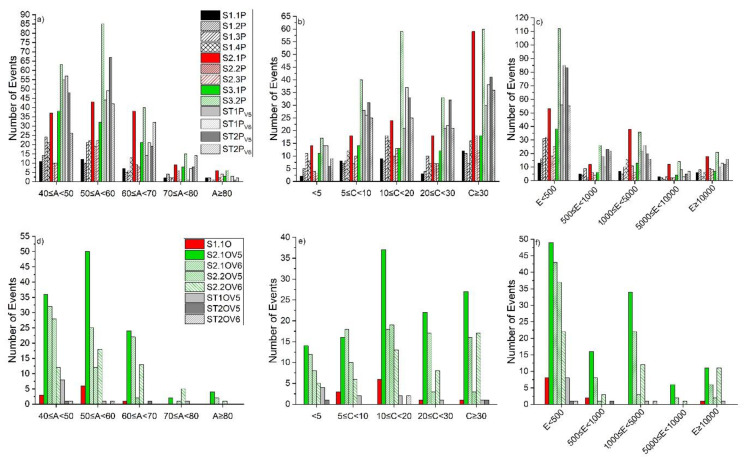
Distribution of AE parameters in the brittle-like (BL) state intervals. (**a**) Amplitude, pine; (**b**) Counts, pine; (**c**) Energy, pine; (**d**) Amplitude, oak; (**e**) Counts oak; (**f**) Energy, oak.

**Figure A1:**
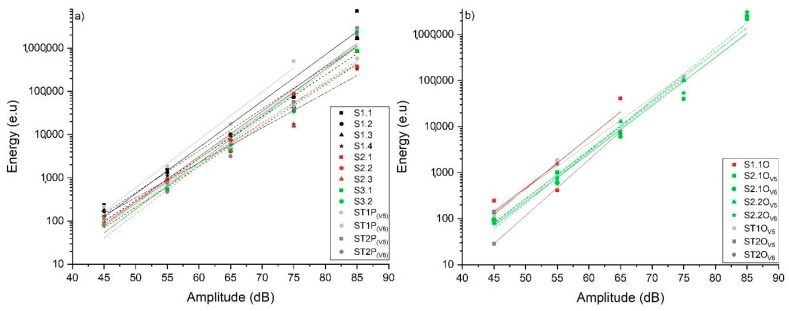
Average values of amplitude classes (<50 dB, 50 dB ≤ A < 60 dB, 60 dB ≤ A < 70 dB, 70 dB ≤ A < 80 dB, and ≥80 dB) vs. corresponding average energy of AE features. (**a**) Pine, brittle state; (**b**) oak, brittle state.

Similarly, the brittle state was analyzed after the grouping of AE events (as described above) emitted during the sequence of load decrease only. The number of emissions within each specific range of amplitude (Figure 6) follows a linear relationship with the energy in a semi-logarithmic plot, as expressed by Equation (3):

(3) $$\begin{array}{l} LogE_{AE}=a+b\times A_{AE}\\ -1.50<a_{Pine}<-3.14\;\;0.08<b_{Pine}<0.11\\ -2.36<a_{Oak}<-3.26\;\;0.10<b_{Oak}<0.11 \end{array}$$

with the intercepts of pine and oak being very similar but having a dependence on the type of surface coating used to protect the wooden sample during the acclimatization period; parallel, the angular coefficient values were almost the same for the two wooden species with an average of 0.10. In the Appendix A (Figure A1 and Table A1), plots and fits are reported for completeness.

The distribution of the amplitude for pine shows a peak, confirmed by all the detections on the different tested samples, for the class including signals between 50 and 60 dB; whereas the distribution of the counts emitted by single AE events displays a linear tendency for both pine and oak. This confirms that which was stated in [2], i.e., the detection of AE single events with higher counts in brittle versus ductile materials. In the time domain, a BL rupture crack shows a combination of phenomena as follows: an increase in the number of events with high-counting signals, while the number of events for low-counting signal is decreasing; and an accelerated growth of the crack [1]. From the analysis of frequency spectra of events collected during the ductile state (Figure 5b,d), differences emerged between pine and oak, since, in the former, the frequency with the highest amplitude falls at about 23 KHz, whereas, for the latter, it falls at 126 KHz. Reference [1] reported that BL fracture, considered to be a severe stage in damage evolution, can be predicted by more compact high frequency peaks and by a decrease in high frequency signals progressing from intermediate damage stages (DL) towards a severe stage (BL), i.e., cell wall cracks [6]. These statements are clearly confirmed by the experimental results shown in Figure 5.

The comparison between the distributions (irrespective of the full-scale) reported in Figure 4 and Figure 6 (displaying the distribution of the selected AE parameters in the ductile and brittle state intervals), clearly shows that a DL behavior exhibits a peak in the AE amplitude distribution at lower values than those detected during BL fracture mechanisms. Similarly, the difference in count distributions indicates a pattern for recognizing DL fracture in wood [1]. In support of the above statements, literature results have assumed that AE behavior with high energy signals in term of energy accumulation during DL fracture indicates slow crack growths that are continuous and steady (e.g., cell separation mechanism) [1,28].

### 3.3. Verification of Latent Emission Sites through the Lifetime within Ductile and Brittle States

During the calibration tests on the simil-CT samples, the pattern highlighted in Figure 3 was macroscopically observed, and showed stages in which the crack progressed very slowly followed by others in which it propagated rapidly. This fact induced the study of the existing relationship between the lifetime spent by the crack progressing while in a DL or BL mode. The presence of two stages of different durability in the fracture process for a stressed material (i.e., initiation stage + crack growth stage) was theorized in [31]. Here, it is detected and analyzed for the first time for wooden materials.

During a DL fracture mechanism, when a local stress is relieved and its associated emissions increases rapidly under raising stress intensity, a crack is prone to advance steadily at a slow propagation rate. This can be expected because during a DL fracture mechanism the catchment area (from which elastic energy can be emitted) is fixed and remains small. In such a situation, sort of “latent emission” sites exist but they are steadily inhibited until a critical (yield strain) value is reached. It is only after reaching this critical value that the crack can abruptly propagate. The “latent emission” sites express the concept defined by [31], as a sort of fracture “quantum” in a DL mechanism that can be transformed into a following BL mechanism (with fast macro crack enlargement) only after a significant number of “initial random micro cracks” cluster.

**Figure 7:**
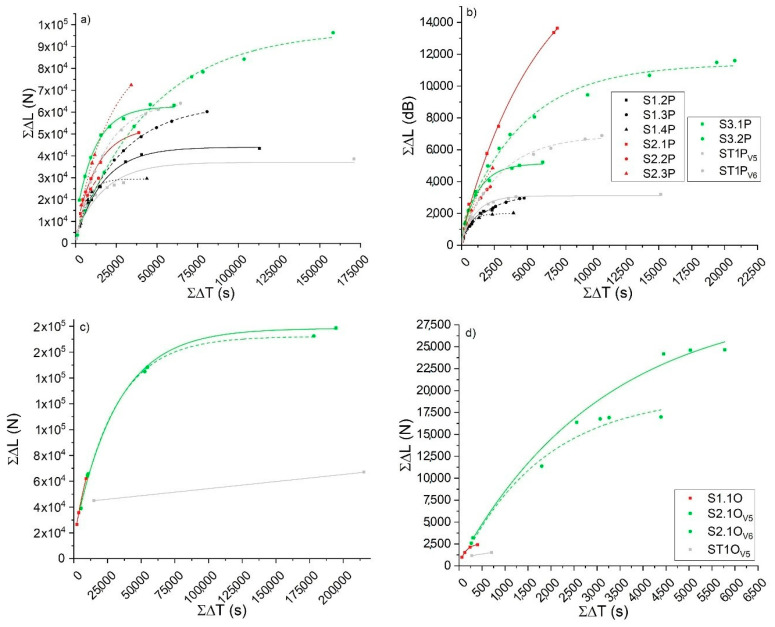
ΣΔT (s) vs. ΣΔL (N) plots of the tested samples and obtained best fits. (**a**) Pine, load increase; (**b**) Pine, load decrease; (**c**) Oak, load increase; (**d**) Oak, load decrease.

Alternatively, in the case of a BL crack growth mechanism, the crack is prone to advance quickly because of the constant progression and renewal of the new catchment area in which elastic energy can be emitted. When considering wood, such sites where the latent emission can be blocked before the crack suddenly propagates undisturbed are the tree rings’ dark areas, i.e., the latewood (see areas in grey and white within Figure 3). On the contrary, the parts where it propagates more extensively and faster are the tree rings’ interspaces (clear areas). These hypotheses have been tested by the analysis of the collected data. The fracture mechanism in the DL state, from the elastic energy emission perspective, can be explained by an almost fixed catchment area or latent emission site. Therefore, from the data analysis, we look to verify that the expected lifetime spent within these sites (i.e., during the load increase sequence), before passing abruptly to the next state, is much longer than the lifetime spent within the catchment area constantly renewed in the case of brittle fracture progression (during the load decrease sequence). The curves of the expected lifetime towards the load intensity (Figure 7) are exponential functions represented by Equation (4) as:

(4) $$\Sigma \Delta L=L_{0}-A\times \exp\left(R_{0}\times \Sigma \Delta t\right)$$

where ΣΔ*L* is the cumulative load (expressed in Newton) during increase and decrease stages; *L*_0_ is a detectable load offset for which there are no acoustic emissions; *A* is an initial value (N); *R*_0_ is a constant rate of survival (s^−1^); and ΣΔ*t* (s) is the total service life, i.e., the time in which the sample survived to the total load up to the total failure.

The equations of the relationships as detected by the AE during BL fracture mechanism (i.e., quick crack propagation), as well as the total expected lifetime for both the ductile and brittle state, are reported in Table 2 (see Appendix A (Table A2) for equations related to the ductile state).

The results obtained for all the simil-CT samples confirm that a ductile fracture mechanism spent more time in latent emission sites. Although the observed very high load jump levels as compared with the brittle fracture mechanism (i.e., ratio ranging between 5 and 12 times), during these lifetimes the crack propagation is small, as shown in Figure 3. In addition, there is a clear difference in the two wooden species behavior. The pine survives for almost 2500 s before completely cracking under the BL fracture mechanism with a distinction in the rank of total load decrease. The oak is either faster in reaching comparable load values, as in the case of the uncoated (ST1O) or the S1.1O (CS + Paraloid 40%) samples, or it is slower and it achieves higher ranks (as in the case of the tar-coated samples S2.1O and S2.2O).

### 3.4. AE Characteristic Calibration Curves of Brittle States

Once the methodological approach for the data analysis consisting of separating AE events occurring during ductile and brittle states was verified as sound, the characteristic calibration curves for treated and untreated simil-CT samples of pine and oak were derived for BL fracture mechanisms, i.e., during load decrease stages only.

**Figure 8:**
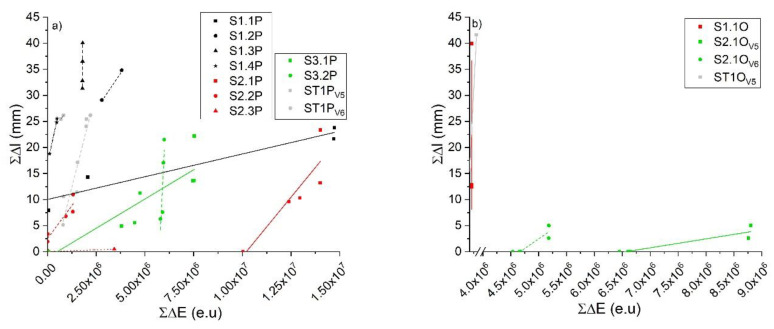
Experimental values and fracture mechanism calibration curves obtained for the brittle-like states (BL) of specimens listed in Table 1 and Table 4. (**a**) Pine; (**b**) Oak.

The calibration curve is a best fit equation which allows AE data interpretation with respect to the extent of fracture in the sample; in this case, the primary metric is the sum of AE energy (A.U.), to be correlated with the total crack length propagation measured using a separate technique (i.e., a camera). The BL state calibration curves are visualized in Figure 8 for the treated and untreated pine (Figure 8a) and oak samples (Figure 8b). The difference between the values obtained through the analysis of the collected images and those calculated using the calibration curves was estimated as ratio (i.e., Equation (5)) (reported in Table 3):

(5) $$FR=\frac{\Sigma l_{AE}}{\Sigma l_{Camera}}$$

The obtained values provide information on the detection quality of the AE in term of elastic energy released during the BL fracture mechanism. If the factor ratio (*FR*) is 1 the detection quality is optimal, as theoretically all the energy emitted during the fracture is detected as AE events. On the other hand, if the *FR* decreases to 0.75, almost 75% of the total energy is emitted as elastic energy and if it is 0.5 only the 50%. The experimental results show that through the BL fracture mechanism calibration curve it is possible to achieve a 0.71 < *FR* < 1.01, effectively approximating the true length of the crack propagation measured from collected images.

Table 4 reports all the BL calibration curves, an objective of this work, that are meaningful fits to estimate crack propagation from AE energy data detected during laboratory tests or structural health monitoring campaigns. In fitting these curves for the load decrease stages, the first point was not considered as suggested by [32] because of the effect of the loading machine and the specimen geometry. All these fits show the linear relation that exists between the maximum energy released and the maximum distance moved by the crack through load jumps. The linear fits have a 0.78 < R^2^ < 0.99 which reflects the possibility to achieve *FR* values close to or lower than 1. The physical interpretation of these calibration curves is that they represent the energy values required to form the new cracked surfaces and to deform the surface layers as the crack propagates. Table 4 also reports the maximum AE detected energy, the maximum fractured area calculated using the BL calibration equations, and the ratio of the sum of AE energy (Σ*E_AE_*) over the maximum fractured area as calculated by the collected images (Σ*A*). This ratio can be interpreted as a “dynamic surface energy value” (DSV) (Equation (6)) as:

(6) $$DSV=\frac{\Sigma E_{AE}}{\Sigma A}$$

**Figure 9:**
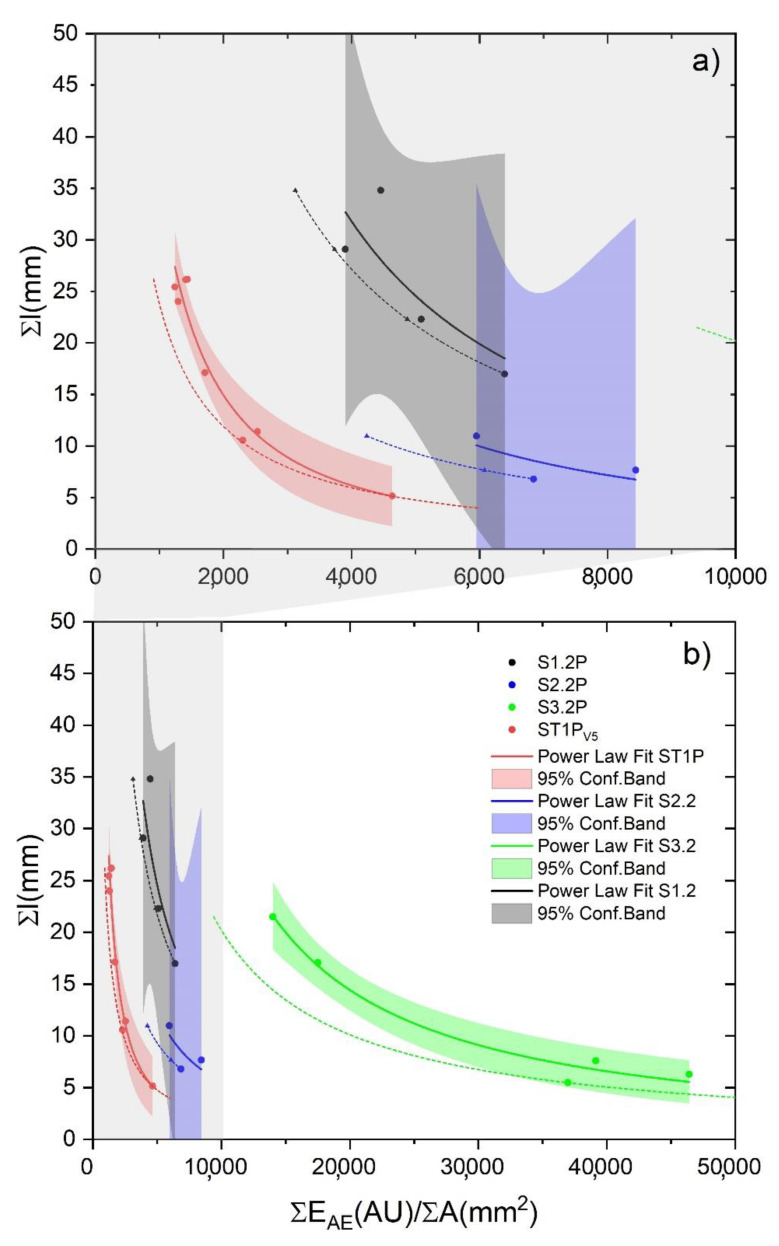
(**a**) Zoomed details of the grey area in (b) for DSV values up to 10,000. (**b**) Dynamic surface energy value (DSV) vs. maximum crack propagation relationship for the four types of tested pine samples, i.e., ST1P (uncoated, red dots and lines), S1.2P (Renaissance Wax, black dots and lines), S2.2P (CS + Paraloid, blue dots and lines), and S3.2P (tar, green dots and lines). Full lines, power law best fits from experimental data; short dashed lines, theoretical loci of equilibrium; shaded areas, 95% confidence band for experimental data.

DSV is an interesting value which, if analyzed step by step during each load drop for a specific tested sample, can explain the dynamic of the crack propagation. Figure 9, showing the DSV versus the maximum allowed crack propagation, is an example of this type of analysis.

The tested samples reported in this plot, all vary with a simple power-law relationship which follows Equation (7) as:

(7) $$\Sigma l=a\times {\left(\frac{\Sigma E_{AE}}{\Sigma A}\right)}^{b}$$

where Σ*E_AE_* is the sum of all the AE energy emitted during load drops, Σ*l* is the total crack propagation length, and Σ*A* is the total fractured area as observed using the camera, while *a* is the coefficient of the power law and *b* is the power (see Table 5).

The meaning of this relation, as stated by the theoretical approach presented in [28], is that of a locus of equilibrium (LoE) of the energy surface rate for crack initiation and arrest which follows Equation (8):

(8) $$\left(\frac{1}{2}\times \Sigma l_{i}\times E_{AE}{}_{initiation,i}\right)$$

The AE energy that is possible to record constitutes the kinetic energy propagated in a stress wave through the specimen. The distance traveled by the crack through load decrease steps during a BL fracture mechanism depends on the difference expressed in Equation (9):

(9) $$\Delta l_{i}=\left(\frac{1}{2}\times \Sigma l_{i}\times E_{AE}{}_{initiation,i}\right)-\frac{\Sigma E_{AE}}{\Sigma A}$$

When this difference becomes smaller the crack jumps become smaller, as highlighted by our experimental data (colored dots and full lines) in Figure 9. In the same plot, the theoretical locus of equilibrium, as formulated by Radon and Pollock [32], is also calculated starting from our data of *E_AE initiation,i_* (short dash lines).

The brittle mechanism in the crack propagation depends on the capacity of the material to resist crack growth until the $\left(\frac{1}{2}\times \Sigma l_{i}\times E_{AE}{}_{initiation,i}\right)$ value in Equation (9) is notably greater than the DSV. This explains the reason why crack steps of differing lengths are observed in a single sample: only under wider differences in Equation (9) an excess of stored energy exists, and it acts as kinetic energy source for rapid crack growth. For smaller crack steps, a smaller fraction of energy released is available to be radiated as AE. This is consistent with the angular coefficients of the amplitude versus energy semi-log plots, reported in Appendix A, all lower than one. The similarities between the theoretical loci of equilibrium and the experimental observations (short dashed and full line, respectively) demonstrate that once a crack growth has started, surface (initiation) energy is the controlling factor which determines when the crack must stop, rather than the stress intensity. This is confirmed experimentally. In addition, their discrepancy also contains interesting information which is further discussed in the next section.

## 4. Discussion

### Demonstration of the Proven Fluctuation

**Figure 10:**
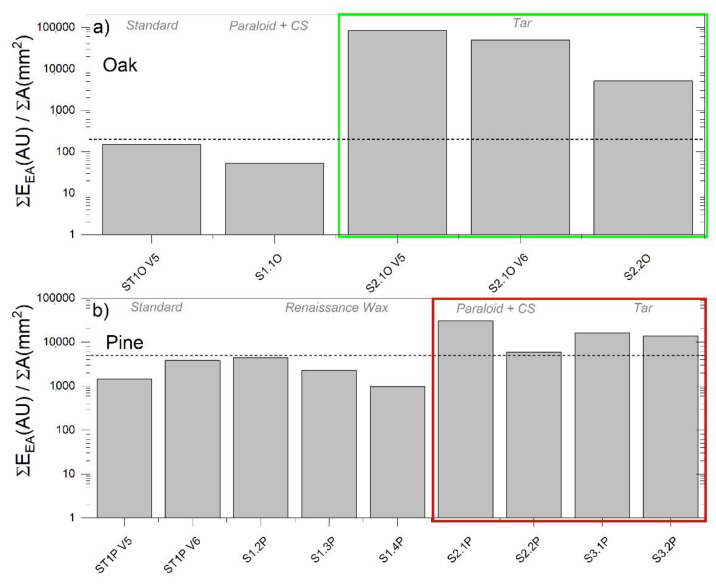
Total DSV (i.e., the ratio between the sum of AE energy (Σ*E_AE_*) over the maximum fractured area (Σ*A*)) for the tested samples. (**a**) From left to right, standard, CS + Paraloid and tar oak samples; (**b**) From left to right, standard, Renaissance wax, CS + Paraloid, and tar pine samples. Green boxes, samples subjected to high constraint (HC) by tar coating and red boxes, samples that showed macro damage during a previous acclimatization period.

DSV, in addition to explaining the crack progression in each sample, also demonstrates the proven concept of fluctuation that was postulated by Michalski in 1993 and later refined [33,34,35] but it has never been proven by experimental data as stated in [36], especially using the AE as proposed in this work. A proven fluctuation can be defined as the pattern of largest RH (or temperature) fluctuations to which a sample/object has been exposed in the past. In this case, the sample/object has already been damaged by climatic factors in the past, and no new damage will occur as long as the current environmental conditions remain lower than the historic extremes. This concept is a sort of immediate risk assessment based on the sample/object history and it has been recently formulated as the European standard (i.e., EN 15757:2010 [37]) within the European Committee for Standardization CEN/TC 346, Conservation of Cultural Heritage. In this standard, a sort of proven fluctuation is indicated to be calculated by assuming RH oscillations in between the 7th and 93rd percentile of the past RH data. However, it is not always possible to record or know the historic RH extremes which a sample/object was subjected to. As anticipated in Section 2, all the samples tested for AE calibration purposes, except the standards, were previously subjected to an acclimatization period in a climate chamber where an abrupt change in RH from 80% to 30% was induced to monitor the AE the reaction. Among the samples, only the samples coming from the pine slices treated with CS + Paraloid (S2.xP) and tar (S3.xP) showed the appearance of macro cracks. Therefore, based on the proven fluctuation concept, assuming the induced RH variation was the only abrupt change to which the samples were subjected to, the calibration tests performed after the acclimatization period should show differences in vulnerability within the set of samples. Samples treated with CS + Paraloid and tar should, thus, have lower vulnerability to crack propagation because of their macro damages or increased constraint that occurred in the past. The DSV, as estimated from the tensile tests, is reported in Figure 9. In the histograms plots, a clear difference is displayed among the samples that were subjected to full mechanical damage at a macro level and those that were not, with the first samples having a higher DSV with respect to the latter ones (green and red squares in Figure 10).

**Figure 11:**
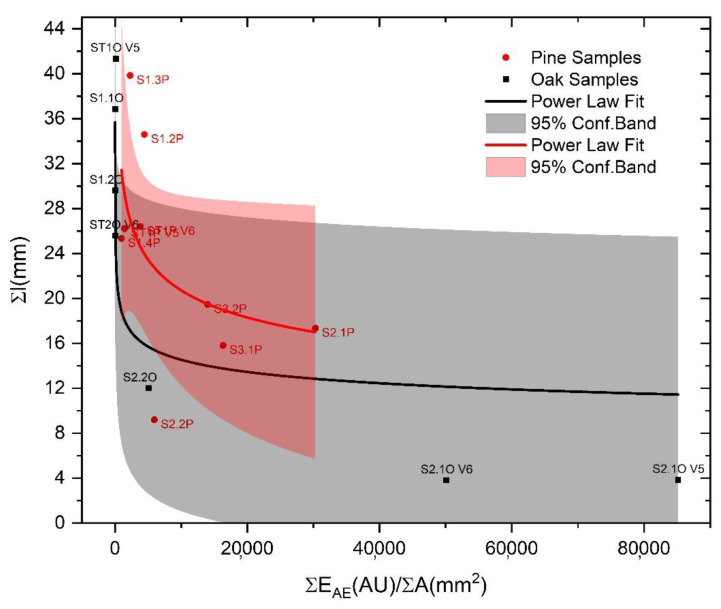
Relationship of the total dynamic surface energy value (DSV) vs. the maximum crack propagation for the two tested wooden species (oak, black dots and line; pine, red dots and line). Lines, power law best fits from experimental data; shaded areas, 95% confidence band for experimental data.

If we compare the results in Figure 9 and Figure 10, in light of the proven fluctuation concept, we observe the following, as shown by the groups of damaged and/or constrained samples coated by tar: (1) a larger discrepancy between the theoretical locus of equilibrium and the experimental best fits and (2) higher values of total DSV. This means the following: (1) The damaged/high constraint (HC) samples should emit less energy to propagate the detected crack steps and (2) a surplus of energy is observed for the damaged/HC samples with respect to the undamaged/low constraint (LC) samples, when subjected to the same climate-induced stress. An explanation for this visible surplus of energy, unable to further propagate a crack (see horizontal discrepancy between the experimental and the theoretical best fit, Figure 9), can be found in the rheological properties of the wood and in the properties of the different types of coating treatments applied on the samples. The wood, with its viscoelastic nature, is subjected to relaxation and creep (i.e., deformation under constant load), whereas, as extensively described in [3,19], the addition of specific coating treatments on its surface (i.e., CS + Paraloid and tar) act as a constraint during its hygric shrinkage during the suddenly induced RH change. This quickly leads to an exceeding of the strain deformation during the acclimatization process. This is what we observed experimentally before the calibration tests, i.e., macro damages developed in the group of pine acclimatized materials highlighted in the red boxes in Figure 10. Results from the calibration tests on acclimatized (damaged/HC and undamaged/LC) and standard reference samples of pine and oak showed different behaviors. We demonstrated that the samples originated from damaged/HC acclimatized materials (i.e., highly vulnerable materials during the acclimatization stage), were less vulnerable in the tensile calibration tests, showing the proven fluctuation behavior. On the contrary, the samples originated by undamaged/LC acclimatized materials were more prone to crack propagation. Figure 11 displays these results assessing the final effects of the fracture tests and shows the total DSV versus the maximum crack propagation of each tested sample. The behavior behind the observed data pattern resembles the “cold-work strain hardening” observable in metallic solids. After cold-work strain hardening, the multiplied and accumulated microscopic dislocations end up interfering with each other, increasing the amount of point defects, and therefore the mechanical resistance. The result is the deformation of the crystalline grains and an increasing of the yield stress with a consequent reduction of the material vulnerability (i.e., the proven fluctuation concept). A sort of strain-hardening behavior for pine and oak is visible on the flatten tail of the power law best fit and it strongly depends both on the species and on the coating treatment. From the experimental data, only samples treated with CS + Paraloid and tar showed this behavior (Sx2.x and Sx3.x), whereas the others, still vulnerable to crack growth during tensile tests, remained in the steep part of the power law fit. In Figure 9, these last samples (e.g., pine treated with Renaissance wax and standard references) are those exhibiting smaller discrepancy with respect to the theoretical expected behavior. This confirms that the climate-induced change suffered by the material treated with the Renaissance wax (pine) did not exceed the past proven fluctuation keeping it safe from cracking during acclimatization. However, at the same time, this treatment also avoided the beneficial strain-hardening mechanism that would have diminished the vulnerability of this set of samples during the tensile calibration tests.

## 5. Conclusions

Due to its hydrophilic nature, wood is particularly sensitive to stresses deriving form RH variation of the surrounding environment. In this study, we proposed to fill a gap in the utilization of non-destructive techniques such as acoustic emission for structural health monitoring purposes. Specifically, a calibration method was proposed in order to fully exploit the potentiality of acoustic emission as an early warning tool for detecting crack propagation in wood materials. The experimental procedure consisted of tensile tests performed on pine and oak simil-CT specimens both uncoated and treated with different chemicals, as well as the monitoring of the process through AE and a digital camera.

The data analysis showed that it is possible to distinguish between the ductile- and brittle-like fracture mechanisms, the latter being the most significant phenomenon to consider for obtaining calibration curves.

Additionally, this study, for the first time, compared the best fit of the DSV obtained from experimental data with the theoretical data, referring to the loci of equilibrium of the energy surface rate for crack initiation and arrest. It turned out that, for each tested sample, experimental data were in good agreement with the expected theoretical values. Major discrepancies between the experimental best fit and the loci of equilibrium were visible for samples cut from slices that suffered macro damages or high constrains during the preliminary acclimatization stage in a climate chamber, experiencing an abrupt RH drop off from 80 to 30%.

Finally, the mechanical behaviors of acclimatized (damaged/HC and undamaged/LC) and standard reference samples of pine and oak, kept at room conditions for the entire experimental period, were compared. The results indicate that different vulnerability degrees can be individuated depending on the possibility that damaged materials have achieved a sort of strain-hardening limit. The achievement of this condition is compatible with the proven fluctuation concept.

The promising results reported herein represent a good starting point for future deep investigations on this promising research topic.

## Figures and Tables

**Table 1:** Dimensions of the simil-CT pine and oak samples. Samples marked with an asterisk have been considered for calibration curve elaboration. P, pine; O, oak; (V5), vallen sensor number 5. In the sample nomenclature, the meaning of the characters are as follows: S, sample; 1, 2, and 3, treatment type, i.e., Reinnassence wax, CS + Paraloid, and tar; 1 to 4, number of the tested sample with that specific treatment; last character P or O, pine and oak; H, height of the specimen; B, thickness of the specimen; W, length of the specimen. * Samples with BL calibration curves.

Sample	Treatment	2H (mm)	B (mm)	W (mm)
S1.1P	Microcrystalline wax	35.6	19.7	54.0
*S1.2P	Microcrystalline wax	35.5	24.6	64.2
*S1.3P	Microcrystalline wax	35.5	19.7	81.3
*S1.4P	Microcrystalline wax	35.5	19.7	50.5
*S2.1P	CS + Paraloid 40%	35.2	19.8	63.0
*S2.2P	CS + Paraloid 40%	35.5	20.2	50.0
S2.3P	CS + Paraloid 40%	35.3	19.7	78.5
*S3.1P	Tar	35.3	2.08	63.2
*S3.2P	Tar	35.6	19.9	84.0
*ST1P _(V5, V6)_	/	36.8	22.0	69.7
ST2P	/	34.2	21.5	47.3
*S1.1O	CS + Paraloid 40%	35.5	20.7	69.6
S1.2O	CS + Paraloid 40%	35.1	20.4	50.1
*S2.1O _(V5, V6)_	Tar	35.4	20.6	69.3
S2.2O	Tar	35.3	20.9	46.4
*ST1O _(V5, V6)_	/	34.7	20.0	73.8
ST2O	/	34.4	20.2	51.3

**Table 2:** Total expected lifetime for the ductile-like (DL) and brittle-like (BL) state for the simil-CT oak and pine samples and the best fit of lifetime versus load decrease (BL fracture mechanism). V5, Vallen sensor number 5; and V6, Vallen sensor number 6.

Samples	Lifetime (s) vs. Load Decrease (N) Best Fit	DL Lifetime (s)	BL Lifetime (s)
ST1P_(V5)_	$\Sigma \Delta L=3107-2899\times exp\left(-892,569\times 10^{-4}\times \Sigma \Delta t\right)$	29,375	4139
ST1P_(V6)_	$\Sigma \Delta L=6881-6499\times exp\left(-343,101\times 10^{-4}\times \Sigma \Delta t\right)$	64,405	10,670
S1.2P	$\Sigma \Delta L=60+57\times exp\left(1.02283\times 10^{-4}\times \Sigma \Delta t\right)$	112,820	2320
S1.3P	$\Sigma \Delta L=76+64\times exp\left(4.32901\times 10^{-5}\times \Sigma \Delta t\right)$	80,716	4769
S1.4P	$\Sigma \Delta L=38+76\times exp\left(1.71289\times 10^{-4}\times \Sigma \Delta t\right)$	43,754	3950
S2.1P	$\Sigma \Delta L=1292+1175\times exp\left(2.20514\times 10^{-5}\times \Sigma \Delta t\right)$	15,259	7295
S2.2P	$\Sigma \Delta L=4537-4433\times exp\left(-7.36839\times 10^{-4}\times \Sigma \Delta t\right)$	14,086	2185
S3.1P	$\Sigma \Delta L=5133-4751\times exp\left(-7.90557\times 10^{-4}\times \Sigma \Delta t\right)$	9,736,926	6173
S3.2P	$\Sigma \Delta L=11,389-10,235\times exp\left(-2.13416\times 10^{-4}\times \Sigma \Delta t\right)$	158,127	20,770
ST1O_(V5)_	ΣΔL=959+0.78815×ΣΔt	215,395	703
S1.1O	ΣΔL=2577−2537×exp(−0.00709×ΣΔt)	9181	404
S2.1O_(V5)_	$\Sigma \Delta L=30,341-29,975\times exp\left(-3.17721\times 10^{-4}\times \Sigma \Delta t\right)$	194,656	5778
S2.1O_(V6)_	$\Sigma \Delta L=19,569-19,803\times exp\left(-5.49419\times 10^{-4}\times \Sigma \Delta t\right)$	178,220	4390

**Table 3:** Dimension of crack lengths as observed by the camera (true crack) and by the AE system (calculated crack). The factor ratio *FR* (pure number) is representative of the detection quality of the AE in terms of elastic energy released during the brittle fracture.

Samples	Σ*l_Camera_* (mm)	Σ*l_AE_* (mm)	*FR*
ST1P_(V5; V6)_	26.18	26.20	1.01
S1.2 P	34.80	34.59	0.99
S1.3P	40.02	39.82	1.00
S1.4P	25.55	25.32	0.99
S2.1P	23.35	17.33	0.74
S2.2P	10.97	9.19	0.84
S3.1P	22.16	15.80	0.71
S3.2P	21.50	19.44	0.90
ST1O_(V5)_	41.59	41.33	0.99
S1.1O	39.91	36.84	0.92

**Table 4:** BL calibration curves equation to correlate the total emitted *E_AE_* with the crack propagation during BL fracture mechanism. The maximum AE energy (A.U.), fractured area (mm^2^) and the “dynamic surface energy value” (DSV) are also reported.

Samples	Calibration Curve	R^2^	Σ*E_AE_* (AU)	ΣA_AE_^1^ (mm^2^)	Σ*E_AE_*/Σ*A* ^1^ (mm^2^)
ST1P _(V5)_	$\Sigma \Delta l_{i}=21.46+5.73\times 10^{-6}\times \Sigma \Delta E_{i}$	0.99	828,199	576.47	1438
ST1P _(V6)_	$\Sigma \Delta l_{i}=-5.97+1.47\times 10^{-5}\times \Sigma \Delta E_{i}$	0.92	2,207,696	580.57	3833
S1.2P	$\Sigma \Delta l_{i}=13.48+5.53\times 10^{-6}\times \Sigma \Delta E_{i}$	0.99	3,815,349	850.85	4457
S1.3P	$\Sigma \Delta l_{i}=-1632.18+9.27\times 10^{-4}\times \Sigma \Delta E_{i}$	0.99	1,803,550	784.51	2288
S1.4P	$\Sigma \Delta l_{i}=16.92+1.7\times 10^{-5}\times \Sigma \Delta E_{i}$	0.99	490,119	498.78	974
S2.1P	$\Sigma \Delta l_{i}=-46.50+4.56\times 10^{-6}\times \Sigma \Delta E_{i}$	0.79	14,004,800	343.20	30,297
S2.2P	$\Sigma \Delta l_{i}=2.52+5.06\times 10^{-6}\times \Sigma \Delta E_{i}$	0.87	1,319,162	185.67	5951
S3.1P	$\Sigma \Delta l_{i}=-1.11+2.25\times 10^{-6}\times \Sigma \Delta E_{i}$	0.83	7,535,067	328.71	16,346
S3.2P	$\Sigma \Delta l_{i}=-461.46+8.03\times 10^{-5}\times \Sigma \Delta E_{i}$	0.78	5,991,167	386.95	14,004
ST1O _(V5)_	$\Sigma \Delta l_{i}=15.17+2.08\times 10^{-4}\times \Sigma \Delta E_{i}$	0.99	125,739	826.52	151
S1.1O	$\Sigma \Delta l_{i}=-638.48+1.52\times 10^{-2}\times \Sigma \Delta E_{i}$	0.83	44,429	762.69	54
S2.1O _(V5)_	$\Sigma \Delta l_{i}=-11.27+1.72\times 10^{-6}\times \Sigma \Delta E_{i}$	0.86	8,802,952	79.17	85,147
S2.1O _(V6)_	$\Sigma \Delta l_{i}=-34.50+7.39\times 10^{-6}\times \Sigma \Delta E_{i}$	0.82	5,181,002	78.44	50,113

^1^ ΣA_AE_, fractured area as calculated using the calibrations curves and the known thickness of the samples; Σ*A*, fractured area as obtained by the camera measures.

**Table 5:** Values of the coefficient (**a**) and power (**b**) in the best fit power laws reported in Figure 9 and of the theoretical locus of equilibrium (LoE) (see below for the four types of tested pine samples, i.e., ST1P (uncoated, red dots and lines), S1.2P (Renaissance Wax, black dots and lines), S2.2P (CS + Paraloid, blue dots and lines), and S3.2P (tar, green dots and lines). Damage and undamaged or low and high constraint notes are also reported.

Samples	a	b	LoE Value ^1^	Note
ST1P _(V5)_	237,031.08	−1.27	11,890.05	-
S1.2P	461,118.66	−1.16	54,304.96	LC ^1^
S2.2P	211,429.60	−1.15	23,251.01	crack
S3.2P	1,098,340.00	−1.13	101,009.39	crack

^1^ LoE (locus of equilibrium) value as calculated, point by point, with Equation (8). LC, low constraint.

## Appendix A

**Table A1:** Equations of best fit curves shown in Figure A1a,b, referring to the brittle state of tested specimens.

Samples	Fit	R^2^
ST1P _(V5)_	Log E_AE_ = −2.76 + 0.11 × A_AE_	0.99
ST1P _(V6)_	Log E_AE_ = −2.28 + 0.09 × A_AE_	1.00
S1.2P	Log E_AE_ = −2.18 + 0.10 × A_AE_	0.99
S1.3P	Log E_AE_ = −2.35 + 0.10 × A_AE_	0.96
S1.4P	Log E_AE_ = −1.50 + 0.08 × A_AE_	0.98
S2.1P	Log E_AE_ =−2.79 + 0.10 × A_AE_	0.98
S2.2P	Log E_AE_ = −2.13 + 0.09 × A_AE_	0.99
S3.1P	Log E_AE_ = −3.14 + 0.11 × A_AE_	0.98
S3.2P	Log E_AE_ = −2.49 + 0.10 × A_AE_	0.98
ST1O _(V5)_	Log E_AE_ = −2.36 + 0.10 × A_AE_	0.99
S1.1O	Log E_AE_ = −2.91 + 0.11 × A_AE_	0.83
S2.1O _(V5)_	Log E_AE_ = −2.85 + 0.10 × A_AE_	0.98
S2.1O _(V6)_	Log E_AE_ = −3.26 + 0.11 × A_AE_	0.99

**Table A2:** Total expected lifetime for the ductile and brittle state for the simil-CT oak and pine samples and the best fit of lifetime versus load increase (DL fracture mechanism).

Samples	Best Fit of Lifetime (s) vs. Load Increase (N)	DL Lifetime (s)	BL Lifetime (s)
ST1P _(V5)_	$\Sigma \Delta L=37,043-32,847\times exp\left(-5.50937\times 10^{-5}\times \Sigma \Delta t\right)$	29,375	4139
ST1P _(V6)_	$\Sigma \Delta L=64,821-63,621\times exp\left(-5.77628\times 10^{-5}\times \Sigma \Delta t\right)$	64,405	10,670
S1.2P	$\Sigma \Delta L=43,949-42,348\times exp\left(-5.83239\times 10^{-5}\times \Sigma \Delta t\right)$	112,820	2320
S1.3P	$\Sigma \Delta L=63,558-58,862\times exp\left(-3.46434\times 10^{-5}\times \Sigma \Delta t\right)$	80,716	4769
S1.4P	$\Sigma \Delta L=29,430-34,500\times exp\left(-1.70288\times 10^{-4}\times \Sigma \Delta t\right)$	43,754	3950
S2.1P	$\Sigma \Delta L=53,039-47,431\times exp\left(-7.46068\times 10^{-5}\times \Sigma \Delta t\right)$	15,259	7295
S2.2P	$\Sigma \Delta L=35,931-36,291\times exp\left(-1.27287\times 10^{-4}\times \Sigma \Delta t\right)$	14,086	2185
S3.1P	$\Sigma \Delta L=62,558-51,992\times exp\left(-8.48307\times 10^{-5}\times \Sigma \Delta t\right)$	9,736,926	6173
S3.2P	$\Sigma \Delta L=97,544-94,015\times exp\left(-2.09852\times 10^{-5}\times \Sigma \Delta t\right)$	158,127	20,770
ST1O _(V5)_	ΣΔL=43,312+0.10969×ΣΔt	215,395	703
S1.1O	$\Sigma \Delta L=121,423-110,333\times exp\left(-6.73096\times 10^{-5}\times \Sigma \Delta t\right)$	9181	404
S2.1O _(V5)_	$\Sigma \Delta L=178,482-161,431\times exp\left(-3.13564\times 10^{-5}\times \Sigma \Delta t\right)$	194,656	5778
S2.1O _(V6)_	$\Sigma \Delta L=172,256-157,515\times exp\left(-3.42184\times 10^{-5}\times \Sigma \Delta t\right)$	178,220	4390

## References

[1] Fukun X., Houran W., Gang L. (2019). Study on Multiparameter Precursory Information Identification of the Fracture of Yellow Sandstone. Adv. Civ. Eng..

[2] Pollock A.A., Tom P. (2010). Material Brittleness and the energetics of acoustic emission. Experimental Mechanics on Emerging Energy Systems and Materials, Proceedings of the 2010 Annual Conference on Experimental and Applied Mechanics.

[3] (2020). Standard Terminology for Nondestructive Testing, E 1316.

[4] Schniewind A.P., Quarles S.L., Lee S.H. (1996). Wood fracture, acoustic emission, and the drying process Part 1. Acoustic emission associated with fracture. Wood Sci. Technol..

[5] Strojecki M., Colla C., Łukomski M., Gabrielli E. (2013). Kaiser effect in historic timber elements. Eur. J. Wood Wood Prod..

[6] Baensch F., Sause M.G.R., Brunner A.J., Niemz P. (2015). Damage evolution in wood—Pattern recognition based on acoustic emission (AE) frequency spectra. Holzforschung.

[7] Kawamoto S., Williams R.S. (2002). Acoustic Emission and Acoustic Ultrasonic Techniques for Wood and Wood-Based Composites, a Review.

[8] Noguchi M., Ishii R., Fujii Y., Imamura Y. (1992). Acoustic emission monitoring during partial compression to detect early stages of decay. Wood Sci. Technol..

[9] Palma P., Steige R. (2020). Structural health monitoring of timber structures—Review of available methods and case studies. Constr. Build. Mater..

[10] Carpinteri A., Lacidogna G. (2006). Structural Monitoring and Integrity Assessment of Medieval Towers. J. Struct. Eng..

[11] Carpinteri A., Lacidogna G., Manuello A., Niccolini G. (2015). A study on the structural stability of the Asinelli Tower in Bologna. Struct. Control Health Monit..

[12] Bertetto A.M., Masera D., Carpinteri A. (2020). Acoustic Emission Monitoring of the Turin Cathedral Bell Tower: Foreshock and Aftershock Discrimination. Appl. Sci..

[13] DeBaise G.R., Porter A.W., Pentoney R.E. (1966). Morphology and mechanics of wood fracture. Mater. Res. Stand..

[14] Skaar C., Simpson W.T., Honeycutt R.M. (1980). Use of acoustic emissions to identify high levels of stress during oak lumber drying. For. Prod. J..

[15] Ansell M.P. (1982). Acoustic emission from softwoods in tension. Wood Sci. Technol..

[16] Sato K., Fushitani M., Noguchi M. (1984). Discussion of Tensile Fracture of Wood Using acoustic emission, Estimation of tensile strength and consideration of AE generation based on fracture mechanics. J. Jpn. Wood Res. Soc..

[17] Reiterer A., Stanzl-Tschegg S.E., Tschegg E.K. (2000). Mode I Fracture and Acoustic Emission of Softwood and Hardwood. Wood Sci. Technol..

[18] Ando K., Hirashima Y., Sugihara M., Hirao A., Sasaki Y. (2006). Microscopic processes of shearing fracture of old wood, examined using the acoustic emission technique. J. Wood Sci..

[19] de Ferri L., Strojecki M., Bertolin C. Preliminary results on surface treatments on wood. Proceedings of the Florence Heri-Tech.

[20] Bertolin C., de Ferri L., Strojecki M. (2020). Application of the Guggenheim, Anderson, de Boer (GAB) equation to study the impact of sealing treatments on pine wood sorption characteristics. Mater. Des. Process. Commun..

[21] Tronskar J.P., Mannan M.A., Lai M.O. (2003). Application of Acoustic Emission for Measuring Crack Initiation Toughness in Instrumented Charpy Impact Testing. J. Test. Eval..

[22] Bertolin C., de Ferri L., Grottesi G., Berto F., Aalto P. (2019). Measuring Protocol in coupling tests on wood using the acoustic emission no-destructive technique. Proceedings of the International Conference on Strcutural Health Assessment of Timber Structures—SHATiS.

[23] Zhang J., Yang S., Hao R., Gu X. (2019). Amplitude attenuation laws of acoustic emission waves in plate structures. Tecnol. E Ing. Mecánicas.

[24] Lukomski M., Strojecki M., Pretzel B., Blades N., Beltran V.L., Freeman A. (2017). Acoustic emission monitoring of micro-damage in wooden art objects to assess climate management strategies. Insight Non-Destr. Test. Cond. Monit..

[25] Łukomski M., Bratasz Ł., Hagan E., Strojecki M., Beltran V.L. (2020). Acoustic Emission Monitoring for Cultural Heritage.

[26] Addali A., Mba D., Mathew J., Ma L., Tan A., Weijnen M., Lee J. (2010). Acoustic Emission Technology for Assessing Gas Void Fraction Levels in Two-Phase Flow. Engineering Asset Management and Infrastructure Sustainability.

[27] Scruby C.B. (1987). An Introduction to Acoustic Emission. J. Phys. E Sci. Instrum..

[28] Baensch F., Zauner M., Sanabria S.J., Sause M.G.R., Pinzer B.R., Brunner A.J., Stampanoni M., Niemz P. (2015). Damage evolution in wood: Synchrotron radiation micro-computed tomography (SRμCT) as a complementary tool for interpreting acoustic emission (AE) behavior. Holzforschung.

[29] Arumugam V., Sajith S., Stanley A.J. (2011). Acoustic Emission Characterization of Failure Mode in GFRP Laminates Under Mode I Delamination. J. Nondestruct. Eval..

[30] Ahn S.H., Nam K.W. (2003). The Determination of J_Ic_ Using the Time-Frequency Analysis Method. J. Test. Eval..

[31] Zhurkov S.N., Kuksenko V.S., Petrov V.A. (1984). Principles of the kinetic approach of fracture prediction. Theor. Appl. Fract. Mech..

[32] Radon J.C., Pollock A.A. (1972). Acoustic Emissions and energy transfer during crack propagation. Eng. Fract. Mech..

[33] Michalski S. (1993). Relative Humidity: A Discussion of Correct/Incorrect Values. Proceedings of the Preprints of the ICOM Committee for Conservation 10th Triennial Meeting.

[34] Michalski S., Bergsma F. (2007). The ideal climate, risk management, the ASHRAE chapter, proofed fluctuations, and toward a full risk analysis model. Proceedings of the Experts’ Roundtable on Sustainable Climate Management Strategies.

[35] Michalski S., Bridgland J. (2014). The Power of History in the Analysis of Collection Risks from Climate Fluctuations and Light. Proceedings of the ICOM Committee for Conservation 17th Triennial Meeting.

[36] Eibl M., Burmester A., Ashley-Smith J., Burmester A., Eibl M. (2013). Learning from history. Historic Indoor Climate Conditions and Climate Control Strategies in Climate for Collections Standards and Uncertainties, Proceedings of the Postprints of the Munich Climate Conference, 7–9 November 2012.

[37] CEN (2010). Conservation of Cultural Property—Specifications for Temperature and Relative Humidity to Limit Climate-Induced Mechanical Damage in Organic Hygroscopic Materials.

